# ﻿Two new species of the genus *Psilalcis* Warren, 1893 (Geometridae, Ennominae, Boarmiini) from Hainan, China

**DOI:** 10.3897/zookeys.1190.115839

**Published:** 2024-01-25

**Authors:** Bo Liu

**Affiliations:** 1 Coconut Research Institute, Chinese Academy of Tropical Agricultural Sciences, Wenchang 571339, China Coconut Research Institute, Chinese Academy of Tropical Agricultural Sciences Wenchang China

**Keywords:** Geometridae, new species, *Psilalcis*, *P.subalbibasis*, *P.subconceptaria*, taxonomy

## Abstract

Two new species, *Psilalcissubalbibasis* Liu, **sp. nov.** and *Psilalcissubconceptaria* Liu, **sp. nov.**, are described from Hainan Island, China. Adult males and females of both species, including their genitalia, are figured and compared to closely related species.

## ﻿Introduction

The genus *Psilalcis*, belonging to the tribe Boarmiini in the subfamily Ennominae of the Geometridae, was established by Warren (1893) with *Tephrosiainceptaria* Walker, 1866 from Flores, Indonesia as its type species. Two other new species, *Psilalcisatrifasciata* Warren and *Psilalcisdentilinea* Warren, both from Sikkim, were also described as members of this new genus in Warren’s article; the former was treated as a synonym of *Parapholodesfuliginea* (Hampson) by Sato (2000); the latter was transferred to *Prochasma* by Prout (1926), primarily based on the presence of the metallic mesothoracic crest. Only a few species and subspecies were included in *Psilalcis* over the following one hundred years (Warren 1899; Inoue 1956, 1964; Sato 1993a, 1993b). Subsequently, Holloway [1994] placed *Paralcis* Warren, 1894 (type species: *Menophraconspicuata* Moore, 1888) as a synonym of *Psilalcis* because of the similar genitalic characters and proposed a broad sense of *Psilalcis* that included the genera *Heterarmia* Warren, 1895, *Polymixinia* Wehrli, 1943, and *Protoboarmia* McDunnough, 1920. He also thought that the genus *Phanerothyris* Warren, 1895 might be referable to *Psilalcis* but had a more distinctive valve structure. In addition, he summarized the characters for the whole group, primarily based on the features of the male and female genitalia, and treated four Bornean species as members of *Psilalcis* with two new species. In the following nearly 30 years, a number of new members, including many newly described species, were added to *Psilalcis* (Sato 1995, 1996, 1998, 1999, 2002, 2008a, 2008b, 2013, 2020, 2023; Inoue 1998; Beljaev and Stüning 2000; Orhant 2001; Sato and Wang 2006, 2016; Stüning 2018). The present *Psilalcis* is a complex with large numbers of species belonging to several different groups on the basis of external characters, and there is evidently much more revisional work to be done.

Recently, two new species, *Psilalcissubalbibasis* sp. nov. and *Psilalcissubconceptaria* sp. nov., were collected from Hainan Island, China; the former is similar in external appearance and genitalia to its close relatives *P.albibasis* (Hampson, 1895), *P.benefica* (Sato, 1993) and *P.sumatrana* Sato, 2013; the latter, together with its close relatives *P.conceptaria* Holloway, 1994, *P.paraceptaria* Sato, 1996 and *P.vietnamensis* Sato, 1996, share unique features of a trifid valve structure and a setose ampulla at the base of a central laminate lobe on the male genitalia, which perhaps can be treated as a separate group. In the present paper, these two new species are described, and their definitive diagnoses are given with respect to closely related species.

## ﻿Materials and methods

All specimens of *Psilalcis* treated herein were collected by light traps on Hainan Island, China and currently are deposited in Coconut Research Institute, Chinese Academy of Tropical Agricultural Sciences, Wengchang, China (**CRICATAS**). For long-term preservation, most of the type specimens of the two new species, including the holotypes, will be transferred to the Institute of Zoology, Chinese Academy of Sciences, Beijing, China (**IZCAS**) and some of the paratypes will be transferred to the Zoologisches Forschungsmuseum Alexander Koenig, Bonn, Germany (**ZFMK**). Terminology for wing venation followed the Comstock-Needham System (Comstock 1918) as adopted for Geometridae by Scoble (1992) and Hausmann (2001), and that of the genitalia was based on Klots (1970) and Skou and Sihvonen (2015). Abdomens were removed and placed in 10% NaOH solution for examination of the genitalia. Genitalia were dissected in 10% alcohol solution and stained with Chlorazol Black E. Photographs of adults were taken with a Nikon D750 camera using a Nikon AF-S Micro 60 mm f/2.8G ED lens. Photos of genitalia were taken with a KUY NICE E31SPM digital camera attached to a Nikon SMZ745T microscope.

## ﻿Taxonomic account

### ﻿ Psilalcis subalbibasis sp. nov.

Taxon classificationAnimaliaLepidopteraGeometridae

53E5BAC4-088F-51A6-9BAA-C6BFAD440457

https://zoobank.org/8E0EAB41-CFAB-4E7F-BA60-D865FDD6F543

**Figures 1–12.**
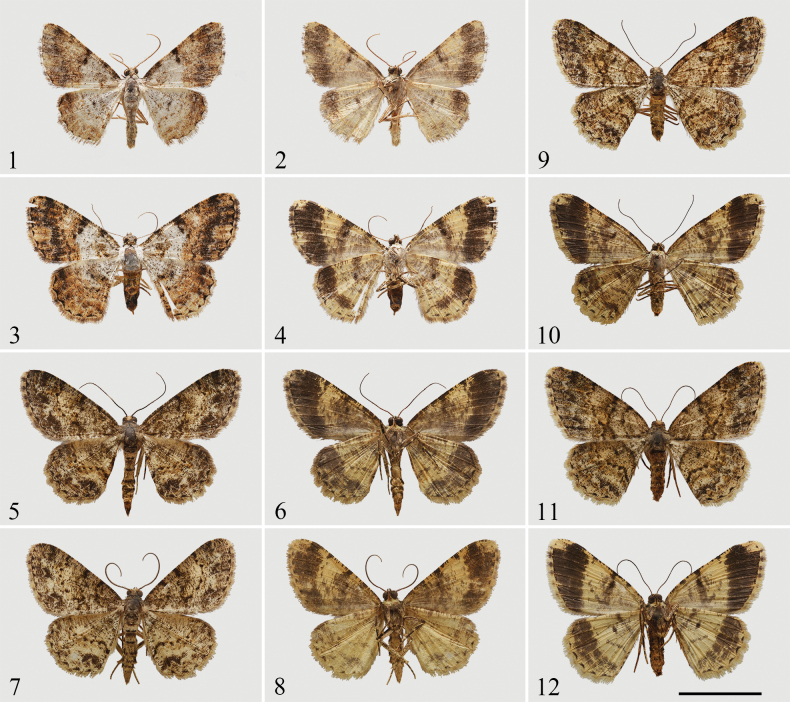
Adults of *Psilalcis* spp. **1–4***Psilalcissubalbibasis* sp. nov. **1** male, holotype, upperside **2** male, holotype, underside **3** female, paratype, upperside **4** female, paratype, underside **5–12***Psilalcissubconceptaria* sp. nov. **5** male, holotype, upperside **6** male, holotype, underside **7** male, paratype, upperside **8** male, paratype, underside **9** female, paratype, upperside **10** female, paratype, underside **11** female, paratype, upperside **12** female, paratype, underside. Scale bar: 1 cm.

**Figures 13, 14.**
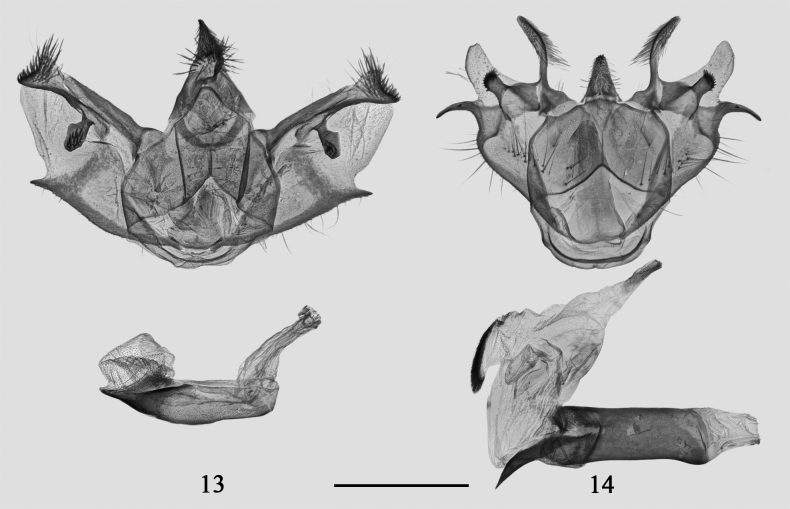
Male genitalia of *Psilalcis* spp. **13***Psilalcissubalbibasis* sp. nov. paratype, gen. prep. no. CRICATAS00112 **14***Psilalcissubconceptaria* sp. nov. paratype, gen. prep. no. CRICATAS00143. Scale bar: 1 mm.

**Figures 15–17.**
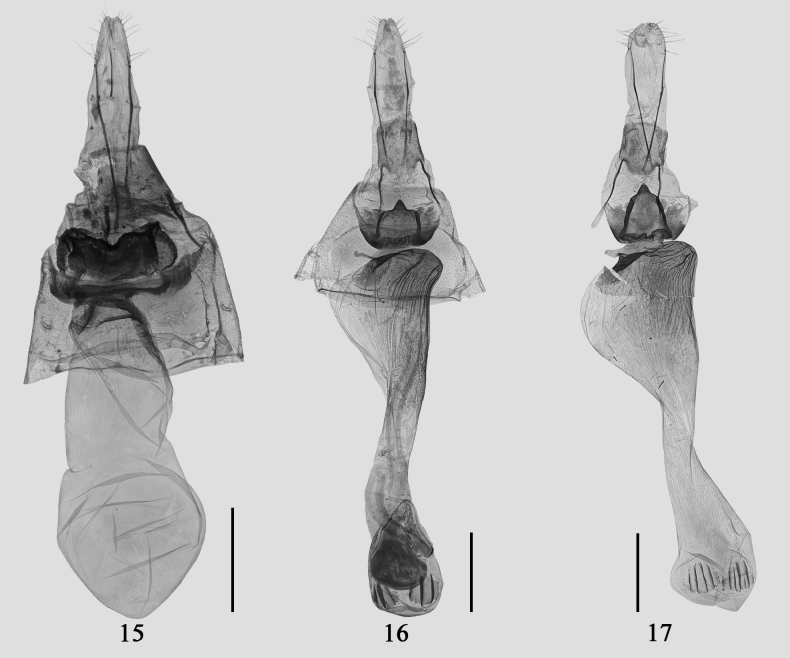
Female genitalia of *Psilalcis* spp. **15***Psilalcissubalbibasis* sp. nov. paratype, gen. prep. no. CRICATAS00113 **16***Psilalcissubconceptaria* sp. nov. paratype, gen. prep. no. CRICATAS00146 **17***Psilalcissubconceptaria* sp. nov. paratype, gen. prep. no. CRICATAS00147. Scale bars: 1 mm.

**Figures 18–21.**
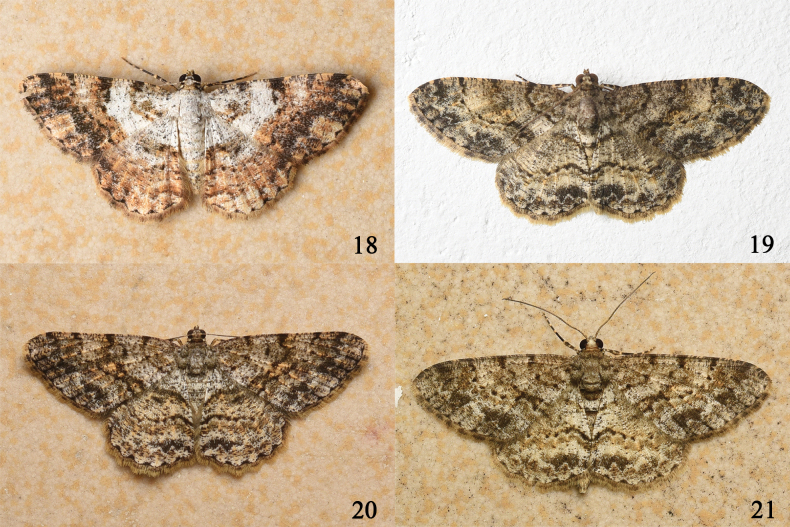
Living specimens of *Psilalcis* spp. **18***Psilalcissubalbibasis* sp. nov. female **19***Psilalcissubconceptaria* sp. nov. male **20***Psilalcissubconceptaria* sp. nov. female **21***Psilalcissubconceptaria* sp. nov. female.

Figs 1–4 , 13 , 15 , 18

#### Type-material.

***Holotype***: ♂, China, Hainan Province, Lingshui, Diaoluoshan, 922 m, 19.VI.2023, Bo Liu leg., gen. prep. no. CRICATAS00112 (CRICATAS, will be transferred to IZCAS in the future). ***Paratype***: 1 ♀, China, Hainan Province, Lingshui, Diaoluoshan, 922 m, 19.VI.2023, Bo Liu leg., gen. prep. no. CRICATAS00113 (CRICATAS, will be transferred to IZCAS in the future).

#### Diagnosis.

*Psilalcissubalbibasis* is very similar in appearance to its close relatives *P.albibasis* (Sato 1999: 37, figs 19, 40; Sato 2020, pl. 25: 18), *P.benefica* (Sato 1993a: 18, pl. 36: 21, fig. 153; Sato 1999: 37; Sato 2013, figs 28, 36; Sato 2020, pl. 25: 17) and *P.sumatrana* (Sato 2013: 250, 251, figs 16–18, 27, 35), all of which have a similar wing pattern of white basal half and reddish-brown terminal half together with a broad dark band. It can be distinguished from its relatives by the following genitalia characters: 1) cucullus crescent-shaped, strongly concave apically in *P.subalbibasis*, triangular and not concave apically in the other three closely related species; 2) setose ampulla narrow at base and broad at apex; and 3) female genitalia with a rather large, sclerotized, uniquely constructed lamella postvaginalis.

#### Description.

Forewing length: male 12.3 mm; female 13.5 mm. Faces more vivid and contrasty in female. ***Head*.** Antennae fasciculate, with moderately long ciliate ventrally in male; filiform in female. Frons not protruding, covered with short scales. Labial palpus curved upwards beyond frons, covered with long, intermingled, dark and fawn scales, third segment not extended in female. Vertex with lamellar, fawn scales, posterior scales erect. Chaetosemata present, small, near eye-margin. ***Thorax*.** Patagia and tegulae with lamellar, white, slightly fawn-colored scales, with longer, pale fawn hair-scales on tegulae only. Prothorax ventrally covered with lamellar, white scales. Legs slender, fawn, chequered black, hind tibia dilated, with a fawn scent brush in male, index of spurs 0-2-4. Forewings with apex angled, termen minutely concave between vein-ends. Fovea present in male, with posterior flexure of the anal vein to accommodate it. Hindwing with apex rounded, termen moderately concave between vein-ends. Wings dark deer-red, mottled dark, with a large white patch at base half, hindwing patch much larger, extending close to submarginal line in male. Antemedial and medial lines dark fawn, faintly visible on forewing, hardly visible on hindwing. Postmedial line fine, dark, slightly sinuous. Discal dot oval, dark, clearly visible, fused with costal patch forming a barred patch on forewing. Submarginal line rather fine, zigzag-shaped, white, faintly visible. Outside of postmedial line bearing a large dark band. Area of apex and between M_3_ and CuA_1_ on forewing without dark colouration. Distal band present only on upper half of hindwing. Marginal line black, inwardly concave. Fringes identical with the ground colour, interspersed with some dark. Underside brownish-yellow, covered with dark streaks. Distal band similar to upperside, but broader and more prominent. Discal dot clearly visible. Medial line more conspicuous in female. ***Venation*.** Forewing: R_1_ and R_2_ coincident; R_1_+R_2_ arising from upper vein of cell, then running almost parallel to the stem of R_3-4_ and R_3-5_; stem of R_3-5_ arising shortly before anterior angle of cell; M_2_ from nearly the middle of the discocellular vein; CuA_1_ from before posterior angle of cell; the base of the anal vein concave downwards. Hindwing: Sc+R_1_ running closely parallel but not anastomosing with upper vein of cell at base; Rs from before anterior angle of cell; CuA_1_ from before posterior angle of cell; 3A present. ***Pregenital abdomen*.** Dorsally scaled white and fawn, scattered with some black scales. Ventrally with pale fawn scales. Setal comb (straight field) of minute setae present on abdominal sternite 3. Tympanal organs moderately sized, without lacinia. A pair of long sterno-tympanal processes present laterally on sternite 1+2, with moderately long free end, reaching the tympanal cavity. Tergite and sternite of segment 7 short, length about 1/2 of width. Tergite and sternite of segment 8 slightly elongate, length approximately equal to width in male.

***Male genitalia*.** Uncus hood-like, base broad, triangular, apex short, strongly sclerotized, curved ventrad at 90 degrees, with strong setae dorsally. Gnathos vestigial, socii not visible. Juxta short, basally broad, apically slightly bifurcated. Saccus rounded, slightly extended. Valvae parallelogram, costa rod-shaped. Cucullus dilated, crescent-shaped, strongly concave apically. Setose digitate ampulla located at the ventral edge of the valve costa, narrow at base, dilated at apex. Valve lamina membranous, central laminate lobe weakly sclerotized. Sacculus sclerotized, distally with a short, slightly inwardly curved spine-like process. Aedeagus short, apex tapering, with a minute spine near the tip. Vesica without cornuti.

***Female genitalia*.** Ovipositor slightly elongated, papillae anales narrow, covered with short setae. Anterior apophyses short, about 2/5 length of posterior apophyses. A thin needle-like sclerite present between the bases of posterior apophyses. Lamella antevaginalis narrow, ribbon-shaped. Lamella postvaginalis rather large, strongly sclerotized; centrally squared, distally concave in the middle; lateral processes expanded, bent dorsad, centrally concave inwards. Posterior part of bursa rather short, with an irregular week of narrow sclerotized band. Anterior part of bursa slightly broader than posterior part, but no clear demarcation visible. Signum absent.

#### Etymology.

This new species, *Psilalcissubalbibasis*, is highly similar to *P.albibasis* (Hampson) in wing pattern and male genitalia.

#### Distribution.

China (Hainan).

### ﻿ Psilalcis subconceptaria sp. nov.

Taxon classificationAnimaliaLepidopteraGeometridae

FBB5DC4B-ACD7-53AB-B0C8-653902958579

https://zoobank.org/DDCF3EEE-FE44-41C3-99E2-B1E0A18A68E8

Figs 5–12 , 14 , 16 , 17 , 19–21

#### Type material.

***Holotype***: ♂, China, Hainan Province, Lingshui Li Autonomous County, Diaoluoshan, 922 m, 19.VI.2023, Bo Liu leg. (CRICATAS, will be transferred to IZCAS). ***Paratypes***: 1 ♂, Hainan Province, Qiongzhong Li and Miao Autonomous County, Yinggeling, 496m, 3.III.2023, Bo Liu leg.; 3 ♂ 2 ♀, China, Hainan Province, Wuzhishan City, Wuzhishan, 756 m, 25.III.2023, Bo Liu leg.; 4 ♂ 10 ♀, China, Hainan Province, Lingshui Li Autonomous County, Diaoluoshan, 922 m, 20.IV.2023, Bo Liu leg. gen. prep. no. CRICATAS00147; 5 ♂ 4 ♀, China, Hainan Province, Lingshui Li Autonomous County, Diaoluoshan, 922 m, 10.V.2023, Bo Liu leg. including gen. prep. nos. CRICATAS00143, CRICATAS00146; 2 ♂ 5 ♀, China, Hainan Province, Lingshui Li Autonomous County, Diaoluoshan, 922 m, 19.VI.2023, Bo Liu leg. (CRICATAS, will be transferred to IZCAS and ZFMK)

#### Diagnosis.

*Psilalcissubconceptaria* shares a very similar wing pattern and similar trifid valve structure of the male genitalia with *P.conceptaria* (Holloway [1994: 235], pl. 15: 13, figs 497, 499; Sato 1996, figs 71, 79), *P.paraceptaria* (Sato 1996: 66, 67, figs 47–50, 72, 80), and *P.vietnamensis* (Sato 1996: 66, figs 41–44, 73, 78; Sato 2020, pl. 25: 6, 7). It can be easily distinguished from the other three related congeners by the following genitalia characters: 1) signum located at the anterior of corpus bursae, smaller; 2) costal process elongated, longer than that of the other three relatives; and 3) apex of valve lamina slender, narrower than that of the other three relatives.

#### Description.

Forewing length: male 12.1–14.5 mm; female 13.3–15.0 mm. Wing pattern variable among individuals, usually more vibrant in females. ***Head*.** Antennae fasciculate, with moderately long ciliate ventrally in males; filiform in females. Frons not protruding, covered with short scales, upper half dark, lower half pale brown. Labial palpus curved upwards beyond frons, covered with long, intermingled, dark and brownish scales, third segment not extended. Vertex with lamellar, brownish scales, posterior scales erect. Chaetosemata present, small, near eye-margin. ***Thorax*.** Patagia and tegulae with lamellar, brownish and dark scales, with longer, dark brownish hair-scales on tegulae only. Prothorax ventrally covered with lamellar, brownish scales. Legs slender, yellow, chequered black, hind tibia dilated, with a yellow scent brush in males, index of spurs 0-2-4. Forewings with apex angled, termen minutely concave between vein-ends. Fovea present in males, with posterior flexure of the anal vein to accommodate it. Hindwing with apex rounded, termen moderately concave between vein-ends. Wings brownish, dotted with white and black scales. Postmedial lines of both wings punctuated, sometimes joined in lines, sinuous, black. Medial and postmedial lines of forewing converge below CuA_2_, then separate. Submarginal line very fine, zigzag-shaped, white, faintly visible. Marginal line black, inwardly concave. Distal band serrated, narrow, evident on forewing, only visible near tornus on hindwing. Discal dot small. Fringes colored brownish, interspersed with some black. Underside brownish-yellow, covered with dark streaks. Distal band sometimes absent or not conspicuous on hindwings. ***Venation*.** Forewing: R_1_ and R_2_ coincident; R_1_+R_2_ arising from upper vein of cell, then running close to the stem of R_3-4_ and R_3-5_; stem of R_3-5_ arising from anterior angle of cell; M_2_ from 1/4 of the discocellular vein close to M_1_ at base; CuA_1_ from before posterior angle of cell; the base of the anal vein concave downwards. Hindwing: Sc+R_1_ running closely parallel but not anastomosing with upper vein of cell at base; Rs from before anterior angle of cell; CuA_1_ from before posterior angle of cell; 3A present. ***Pregenital abdomen*.** Abdomen scaled pale brown, scattered with some black scales. Setal comb (straight field) of minute setae present on the third sternite. Tympanal organs moderately sized, without lacinia. A pair of long sterno-tympanal processes present laterally on sternite 1+2, with moderately long free end, reaching the tympanal cavity. Tergite and sternite of segment 8 strongly elongate in males, length nearly twice the width.

***Male genitalia*.** Uncus hood-like, short, weakly curved ventrally, dorsally with short setae. Gnathos and socii absent. Juxta tongue-like, broad at base, slightly pointed at tip. Saccus rounded, slightly extended. Valvae trifid, costal process elongate, cucullus vestigial. Setose ampulla located at the centre of valve laminate lobe. Apex of sacculus bearing a long, strongly curved spine. Valve lamina membranous, distally elongated, central laminate lobe sclerotized. Aedeagus stout, apex with a curved, slender spine, vesica with a cluster of needle-like cornuti on a lateral lobe.

***Female genitalia*.** Ovipositor slightly elongated, papillae anales narrow, covered with short setae. Anterior apophyses short, about 3/5 length of posterior apophyses. The needle-like sclerite between the bases of posterior apophyses absent. Lamella antevaginalis narrow, ribbon-shaped. Lamella postvaginalis very large, centrally triangular, distally triangularly convex at the centre, lateral processes extended, slightly curved dorsad. Posterior part of bursa much narrower than anterior part, rather short, membranous. Anterior part of bursa elongated, posteriorly projected at both sides, with sclerotized corrugations, centrally with a constriction, anteriorly bearing a pair of small, circular, opposed sclerotized patches, with three to five longitudinal ridges.

#### Etymology.

The specific name, *subconceptaria*, is derived from its closely related species, *P.conceptaria*.

#### Distribution.

China (Hainan).

## Supplementary Material

XML Treatment for Psilalcis subalbibasis

XML Treatment for Psilalcis subconceptaria

## ﻿Additional information

### Author ORCID

Bo Liu https://orcid.org/0009-0008-7003-4659

### Data availability

All of the data that support the findings of this study are available in the main text.
